# Self-generated off-line memory reprocessing on different layers of a hierarchical recurrent neuronal network

**DOI:** 10.1186/1471-2202-12-S1-P162

**Published:** 2011-07-18

**Authors:** Jenia Jitsev

**Affiliations:** 1 Max-Planck-Institute for neurological research, 50931 Cologne, Germany

## 

Memory traces in the cortex are embedded into a scaffold of feed-forward and recurrent connectivity of the hierarchically organized processing pathways. Strong evidence suggests that consolidation of the memory traces in such a memory network depends on an off-line reprocessing done in the sleep state or during restful waking. It remains largely unclear, what plasticity mechanisms are involved in this consolidation process and what changes are induced at what sites in the network during memory reprocessing in the off-line regime.

This study focuses on functional consequences an off-line reprocessing has in a hierarchical recurrent neuronal network that learns different person identities from natural face images in unsupervised manner [1]. Due to the inherently self-exciting, but competitive winner-take-all-like unit dynamics, the two-layered network is able to self-generate sparse activity even in the absence of external input in an off-line regime. In this regime, the network reactivates the memory traces established during preceding on-line learning.

Remarkably, this off-line memory replay turns out to be highly beneficial for the network recognition performance [2]. The benefit is articulated after the off-line regime in a strong boost of identity recognition rate on the alternative face views to which the network has not been exposed during learning. Performance of both network layers is affected by the boost. Surprisingly, the positive effect is independent of synapse-specific plasticity, relying completely on a synapse-unspecific mechanism of homeostatic activity regulation. This homeostatic mechanism tunes network unit excitabilities, equalizing the excitability levels within the network layers during the off-line reprocessing and causing the performance improvement when the network is back in the on-line regime.

Performing excitability equalization for the lower and the higher network layers in separate, it becomes possible to dissociate the contribution of both layers to the positive effect observed after the off-line reprocessing. Equalizing the excitability levels on only one of both layers boosts the network recognition performance, independent of whether the equalization is made on the lower or on the higher layer. The excitability equalization on the higher layer has hereby a slightly stronger effect on network performance. The full boost however is achieved only if both layers are simultaneously processed via excitability equalization. Interestingly, the full effect cannot be simply explained by adding up the separate contributions of each layer, indicating that there is a substantial synergetic interaction between both in achieving the improvement after the off-line memory reprocessing. These findings suggest that all layers of the network hierarchy contribute their distinct part to the improvement of network recognition performance if affected by the off-line reprocessing, which provides interesting hints how off-line memory reprocessing may act on the hierarchically organized pathways in the brain during the states of sleep or restful waking.
